# Plasmonic Interferometers as TREM2 Sensors for Alzheimer’s Disease

**DOI:** 10.3390/bios11070217

**Published:** 2021-07-01

**Authors:** Dingdong Li, Rachel Odessey, Dongfang Li, Domenico Pacifici

**Affiliations:** 1 School of Engineering, Brown University, 184 Hope St, Providence, RI 02912, USA; Dingdong_Li@alumni.brown.edu (D.L.); Rachel_Odessey@brown.edu (R.O.); Dongfang_Li@alumni.brown.com (D.L.); 2 Department of Physics, Brown University, 182 Hope St, Providence, RI 02912, USA

**Keywords:** TREM2 sensors, Alzheimer’s disease, plasmonic interferometry, optical biosensor, surface functionalization

## Abstract

We report an effective surface immobilization protocol for capture of Triggering Receptor Expressed on Myeloid Cells 2 (TREM2), a receptor whose elevated concentration in cerebrospinal fluid has recently been associated with Alzheimer’s disease (AD). We employ the proposed surface functionalization scheme to design, fabricate, and assess a biochemical sensing platform based on plasmonic interferometry that is able to detect physiological concentrations of TREM2 in solution. These findings open up opportunities for label-free biosensing of TREM2 in its soluble form in various bodily fluids as an early indicator of the onset of clinical dementia in AD. We also show that plasmonic interferometry can be a powerful tool to monitor and optimize surface immobilization schemes, which could be applied to develop other relevant antibody tests.

## 1. Introduction

Alzheimer’s disease (AD) is a chronic neurodegenerative disorder that affects more than five million Americans and approximately 50 million people worldwide [1]. AD causes loss of memory followed by loss of ability to think and communicate and, finally, loss of life [2]. As it progresses, AD has devastating effects on the ability of subjects to carry out the events of their day-to-day lives and can create significant mental and emotional distress for loved ones whose identities and relationships to the patient are forgotten. AD is one of the costliest disorders to society, costing over a quarter of a trillion dollars in 2017 alone in the United States [3]. Despite the many incentives and the correspondingly tremendous efforts of biopharmaceutical researchers, no disease-modifying therapy is yet available for AD and the drug candidates put forward to treat or prevent the onset of AD symptoms continue to fail in clinical trials [4].

AD is traditionally diagnosed by monitoring subjects’ behavioral changes because it is challenging to diagnose more definitively without an invasive examination of the brain. This inexact method, which has a misdiagnosis rate of up to 45%, contributes to the untenably high attrition rate of drug candidates by creating an incomplete understanding of disease etiology and, as such, a lack of robust and valid biomarkers on the causal path of the disease [5]. Such biomarkers are essential to effective patient care and, specifically, the efficient development of drug treatments because they enable early and more accurate (i) diagnosis and stratification of patients during trial enrollment, (ii) measurement of target engagement and modulation, and (iii) testing of the therapeutic hypothesis in clinical trials that are already extremely long and costly [6].

### 1.1. TREM2 as Biomarker for Early-Onset Detection of AD

Most cases of AD have a complex, highly polygenic architecture [7]. A number of causal genes for AD have been identified in recent years, many of which play important roles in myeloid cells such as microglia, immune cells in the brain [8,9,10]. One such gene is Triggering Receptor Expressed on Myeloid Cells 2 (TREM2) [11,12,13], which senses brain tissue damage due to aging or neurodegeneration by triggering a microglial response aimed at scavenging and clearing brain tissue debris [14,15,16,17]; genetic variants which impair this function also increase the risk of AD more than threefold [18,19]. Recent studies have shown that TREM2 was abnormally elevated 5 years before the expected onset of symptoms in AD patients [13,16,17]. These findings suggest that TREM2 is in the causal path to disease and among the strongest genetic risk factors for AD [20]. Moreover, TREM2 could be used as an effective biomarker for early stage detection of AD [12] and other neurodegenarative diseases [21].

TREM2 may be found in its soluble form in cerebrospinal fluid (CSF) and other bodily fluids, such as saliva [22]. CSF concentrations of soluble TREM2 have been shown to be higher in AD cases than in controls, they correlate with markers of neurodegeneration, and may be used to quantify glial activation in AD [23]. Recently, soluble CSF TREM2 has also been proposed as a surrogate immune biomarker of neuronal injury in Parkinson’s disease [24]. Because of its extremely low concentration in CSF (∼1–5 ng/mL [13,16,17]), TREM2 is conventionally detected and quantified by enzyme-linked immunosorbent assay (ELISA) [25], a well-established and highly sensitive plate-based assay which uses a multilayered format with a labeled secondary antibody. ELISA, while widely in use and highly sensitive, involves a multi-step incubation protocol that usually calls for 2 to 8 h of preparation time for each step. This, together with its stringent washing and blocking protocols and fluorescent labeling steps, makes ELISA a time-consuming assay that is hardly integrable into point-of-care, portable, or multiplexed biosensing platforms.

### 1.2. Plasmonic Interferometry for Sensing Applications

**Figure 1:**
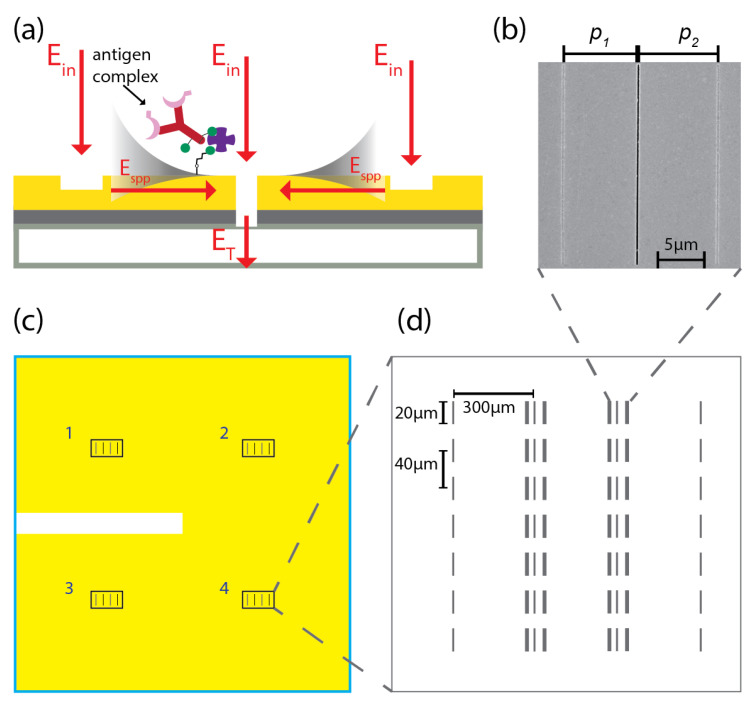
**Design for TREM2 sensor chip based on plasmonic interferometry.** (**a**) Cross-section schematic of the groove-slit-groove (GSG) architecture, which shows a slit flanked by two grooves, from which SPPs are excited by light diffraction and propagate towards the slit aperture, where they interfere and are then transmitted back into free space for far-field detection; diagram includes an example of an antigen complex, further described in Figure 2. The bottom slab represents quartz, the middle titanium, and the top layer gold. (**b**) Scanning electron micrograph (SEM) of a GSG interferometer with $p_{1}=7.65\;\mu$m, $p_{2}=8.15\;\mu$m. (**c**) Schematic of plasmonic interferometer sensor chip layout. The chip contains four nominally identical sensing spots enabling multiplex sensing applications. The yellow area indicates quartz covered by gold and the blank area is an uncoated quartz window used for optical alignment. (**d**) Schematic of a representative active sensing area. Each sensing area contains two columns of single slits and two columns of nominally identical asymmetric GSG interferometers with separation distance of 300 μm. The slit/grooves in each interferometer are ∼20 μm long and, within each column, the distance between two adjacent interferometers is ∼40 μm.

Several optics- and nanostructure-based alternatives to conventional biosensing methods have been developed, including sensing based on magnetic nanoparticles [26], carbon nanotubes and other nanostructures [27], quantum dots [28], and surface plasmon polaritons (SPPs) in gold, especially gold nanoparticles [29]. SPPs are collective oscillations of electrons that may occur when light interacts with the interface between a dielectric and a metal. One promising use of SPPs for biosensing is in plasmonic interferometry, which has been demonstrated to host biosensors with extremely high sensitivity and selectivity [30,31,32,33,34,35,36,37,38,39,40,41,42,43,44]. In this method, SPPs are generated and propagate within a micrometer-scale optical interferometer such as the one in Figure 1a,b: light is incident on a groove-slit-groove (GSG) geometry patterned onto a metal film; optical scattering at the subwavelength-width grooves couples a fraction of the incident light field into SPPs that travel across the surface of the metal, toward the slit. SPPs accrue a propagative phase that depends on the physical distance traveled (that is, the interferometer arm length) and on the SPP refractive index, which is given by [45]:

(1) $${\tilde{n}}_{\mathrm{SPP}}\left(\lambda \right)=n_{\mathrm{SPP}}\left(\lambda \right)+i\kappa_{\mathrm{SPP}}\left(\lambda \right)=\sqrt{\frac{\epsilon_{m}\left(\lambda \right)\epsilon_{d}\left(\lambda \right)}{\epsilon_{m}\left(\lambda \right)+\epsilon_{d}\left(\lambda \right)}},$$

where $n_{\mathrm{SPP}}$ and $\kappa_{\mathrm{SPP}}$ are the real and imaginary parts of ${\tilde{n}}_{\mathrm{SPP}}$, respectively, and $\epsilon_{m}$ and $\epsilon_{d}$ are the complex dielectric functions of the corresponding metal and dielectric material. A small change in $\epsilon_{d}$ (which may be due, for example, to the presence of molecules near the surface) can produce a significant change in the optical path length traveled by an SPP across the surface of the metal [30]. When the counter propagating SPPs arrive at the subwavelength-width slit, they interfere with each other and with the optical beam incident at the slit location before coupling back into free space through the slit. This interference process modulates the light intensity that is transmitted through the slit and detected in the far-field [30,44,46,47]. Since SPPs are highly confined near the metal-dielectric interface, their propagative phase is highly sensitive to the refractive index at the surface. Therefore, the presence of an analyte adsorbed to the surface can be detected even in sub-monolayer concentrations by observing changes in the transmission spectra determined by plasmonic interferometry [30,39,42,43,46]. The sensitivity of a device based on plasmonic interferometry can be enhanced by simply increasing the interferometer arm length, and the signal to noise ratio can be improved by simply changing the geometrical parameters (such as slit/groove width, depth, and length) and the incident wavelength [30,42,44,46].

Compared to more conventional surface plasmon resonance (SPR) sensing platforms, sensors based on plasmonic interferometry (PI) retain high sensitivity and low detection limits whilst providing several advantages, including: (1) broadband (as opposed to single wavelength) operation that allows for spectroscopic capabilities and detection of refractive index changes at multiple wavelengths of interest, simultaneously [30,46]; (2) wide-angle excitation of SPPs (as opposed to the specific angle required to excite the surface plasmon resonance on the metal surface) that enables less stringent alignment requirements [30,43,46] and the use of incoherent light sources, which can also be integrated directly on the sensor surface [42]; (3) smaller sampling volumes and sensing areas, which lead to higher levels of device integration and multiplexing, since the same sensor chip can contain millions of individually addressable devices (over an area of just 1 cm${}^{2}$) that can be used to detect multiple analytes and perform screening on multiple patients at the same time [30,39,42].

Here, we employ plasmonic interferometry to develop and assess a surface functionalization protocol designed to detect TREM2 in solution. Specifically, we (i) monitor each step of the proposed functionalization protocol using the intensity change in the transmitted spectra of plasmonic interferometers and (ii) employ this surface functionalization scheme to detect biological levels of TREM2 in solution.

## 2. Biosensing Chip: Design, Fabrication and Implementation

### 2.1. Fabrication of Biosensing Chip Based on Plasmonic Interferometry

The proposed biosensing chip comprises four arrays of nominally identical GSG plasmonic interferometers that were designed and fabricated with asymmetrical arm lengths ($p_{1}=7.65\;\mu$m, $p_{2}=8.15\;\mu$m) to optimize the device sensitivity to refractive index change caused by TREM2 adsorbed to the sensor surface [30,39,42,44,46]. First, a 4 nm-thick titanium adhesion layer was deposited by electron-beam evaporation onto a previously cleaned 1 mm-thick fused quartz slide followed by a ∼200 nm-thick gold layer. The thickness of the titanium layer was determined experimentally in order to cause strong surface adhesion of the gold layer, which would otherwise tend to delaminate and form blisters if directly deposited on glass. Four sensing spots were milled onto the metal film with a focused ion beam (FIB) using a gallium ion source. Each sensing spot contains two columns of seven nominally identical GSG interferometers and two columns of single slits for the purpose of statistical analysis and normalization, as shown in Figure 1c,d. The distance between two parallel columns and two adjacent interferometers in the same column are 300 μm and 40 μm, respectively. The area of each sensing spot is about 0.2 mm${}^{2}$. Figure 1b shows a scanning electron micrograph of a representative GSG interferometer with left arm length 7.65 μm and right arm length 8.15 μm, within 2% fabrication error. Each groove is approximately 20 μm long, 200 nm wide, and ∼50 nm deep; each slit is 20 μm long, 180 nm wide, and ∼200 nm deep. These parameters were determined by analyzing SEM/FIB cross-sections (not reported). The actual values were chosen to optimize signal-to-noise light transmission ratio, SPP excitation efficiency, amplitude of SPP interference, and overall device sensitivity to refractive index change. Figure 1a shows a schematic illustration of a cross-section of the plasmonic interferometer, with two grooves flanking a slit in order to facilitate incoupling of incident light into SPPs (by optical scattering from each groove) and outcoupling of SPPs back into free space (through the slit).

### 2.2. Surface Functionalization of Optical Biochip

**Figure 2:**
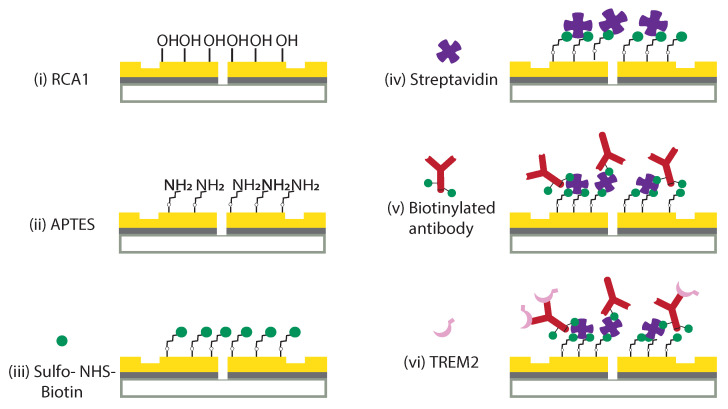
**Surface immobilization protocol for capture of TREM2 in solution.** Chip surface was treated with (**i**) an RCA1 cleaning procedure followed by (**ii**) (3-Aminopropyl)triethoxysilane (APTES) to form an amino-terminated surface. Sulfo-NHS-biotin (sulfo-N-Hydroxysulfosuccinimide biotin) covalently attaches to the amino groups of the surface (**iii**) and subsequently captures streptavidins (**iv**). Finally, the streptavidin functionalized chip is bound by the biotinylated TREM2 antibody (**v**) for sensing of the TREM2 molecule (**vi**). The green dot in (**v**) represents the sulfo-NHS ester of biotin that acts as the biotinylation reagent and allows to form a stable bond between the antibody and the streptavidin already bound to the sensor surface, as reported in (**iv**).

TREM2 antibodies were immobilized on the gold surface by using the protocol shown in Figure 2. The gold chip was first cleaned in an RCA1 solution, a mixture of 20 mL 29% ammonium hydroxide, 20 mL 30% hydrogen peroxide, and 100 mL deionized water (DI water), at 75 °C for 10 min. RCA1 cleaning removes organic residues from the gold surface and forms hydroxyl groups that facilitate silanol groups binding to the surface, as shown in Figure 2i. Next, the cleaned chip was soaked in 8 mL freshly prepared 2% (3-aminopropyl)triethoxysilane (APTES) solution for 1 h at room temperature to form an amino terminated surface, as shown in Figure 2ii. The 2% APTES solution was obtained by serial dilution of 99% APTES (Sigma-Aldrich, Burlington, MA, United States) in DI water. The APTES treatment time and concentration were chosen based on a standard surface plasmon resonance (SPR) functionalization protocol for a gold chip [48,49]. Then, the chip was rinsed thoroughly with DI water to remove loosely adsorbed APTES molecules that hadn’t formed any covalent bonds with the hydroxyl terminated surface.

Next, biotinylation of the surface was achieved by soaking the chip in a 0.5 mg/mL sulfo-NHS-biotin solution (sulfo-N-Hydroxysuccinimide biotin ester sodium salt, Thermo Fisher Scientific, Waltham, MA, United States) for a duration of 2.5 h, followed by rinsing with phosphate buffer solution (PBS) and DI water. The sulfo-NHS-biotin solution was prepared in 0.01 M PBS (pH = 7.4, Sigma-Aldrich) immediately before using. On an amino-terminated gold surface, sulfo-NHS-biotin will covalently attach to the amino groups via ester linkage, as shown in Figure 2iii. Afterwards, the chip was treated with streptavidin by 1 h immersion in a 5 mg/mL fresh streptavidin (Iyophilized powder, Sigma-Aldrich) PBS (0.01 M, pH = 7.4) containing 0.05% Tween 20, which was used to minimize the non-specific binding of streptavidin to the biotinlyated gold surface. The resulting streptavidin-coated chip was subsequently washed three times by washing buffer (0.01 M PBS with 0.05% Tween 20) to remove loosely adsorbed streptavidin molecules and then followed by PBS (0.01 M, pH = 7.4) to wash off the Tween 20 solution, leaving behind a streptavidin-functionalized metal surface, as shown in Figure 2iv.

In order to saturate the sulfo-NHS-biotin binding sites on the APTES-functionalized chip and to form a monolayer of streptavidins and antibodies on the surface, the chip was optically characterized after every hour of chemical treatment until no significant spectral peak shifts were observed in the optical transmission spectra through the GSG interferometers; the concentration ratio of sulfo-NHS-biotin and streptavidin was optimized to achieve a high biotinylated antibody covering rate. Subsequently, the chip was incubated with 0.1 mg/mL biotinylated TREM2 antibody PBS for 2.5 h, as shown in Figure 2v. Finally, human TREM2 biotinylated antibody was purchased from R&D (Cat. #BAF1828, R&D) with 50 μg bovine serum albumin (BSA) per 1 μg as a carrier protein; the yielded solution contains 0.5% BSA in PBS. In sensing experiments, when TREM2 binds to the antibody the resulting chip surface is schematically shown in Figure 2vi.

### 2.3. Optical Characterization of Surface Functionalization Steps with Plasmonic Interferometry

After each functionalization step, the sensor chip was rinsed with DI water to remove unbound molecules/proteins and thoroughly dried under a stream of nitrogen gas to remove any residual water droplets/molecules that might affect the final wavelength shift measurement. This washing and drying protocol was enforced throughout the experiments to make sure that actual protein-protein binding events were measured, which allowed us to better validate the proposed surface functionalization and sensing methods. After surface rinsing and drying, the transmitted intensity of each plasmonic interferometer on the sensor chip was measured. To perform the optical characterization, a plasmonic interferometer sensor chip containing functionalized plasmonic interferometers was placed on the controllable moving stage of a Nikon Eclipse Ti Series inverted microscope. The collimated white light beam from a xenon arc lamp coupled with an optical lens system was focused onto the plasmonic interferometer sensor chip, transmitted through the slit, and collected by an objective lens (0.6 NA, 40×). The collected light was further dispersed by a monochromator and detected by a CCD camera. Transmitted spectra of the single slit accompanying each plasmonic interferometer were taken as well for spectral normalization.

**Figure 3:**
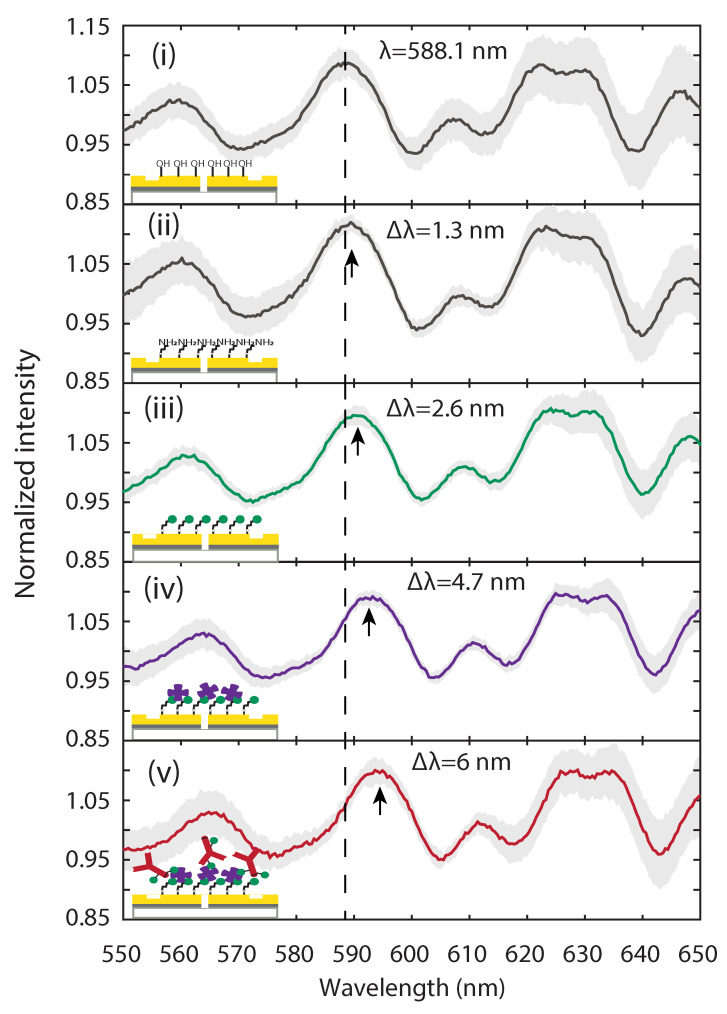
**Tracking functionalization steps through plasmonic interference spectra.** Measured results of transmitted intensity spectra after (**i**) RCA1, (**ii**) APTES, (**iii**) sulfo-NHS-biotin, (**iv**) streptavidin, and (**v**) biotinylated TREM2 antibody treatment. Solid lines represent the mean value of normalized intensity spectra averaged over seven nominally identical GSG interferometers after each functionalization step, as illustrated by the lower left insets. Light gray areas represent standard deviation. The vertical dashed line indicates the position of a representative transmission peak (588.1 nm) that results from constructive SPP interference after RCA1 cleaning. The black arrows mark the wavelength shift (Δλ) in this reference peak as the result of new constructive interference conditions after each functionalization step.

Each solid line in Figure 3 corresponds to the mean value of seven normalized transmitted light intensities after each functionalization step,

(2) $$I_{n,\mathrm{mean}}=\frac{1}{7}\sum_{k=1}^{7}\frac{I_{\mathrm{GSG},k}\left(\lambda \right)}{I_{\mathrm{SS},k}\left(\lambda \right)},$$

where $I_{\mathrm{GSG},k}$ is the background-corrected light intensity transmitted through the *k*th GSG interferometer and $I_{\mathrm{SS},k}$ is the background-corrected light intensity transmitted through the kth single slit, which serves as reference to estimate the light intensity baseline. The light gray shading represents the standard deviation that results from the proposed normalization and averaging procedure.

As shown in Figure 3, several maxima (minima) are observed as a result of constructive (destructive) interference between the counter-propagating SPPs and the original beam at the slit location. To fix the ideas and make it easier to follow the evolution of the various functionalization steps, we choose a specific peak as a reference, although the shift caused by the addition of reagents that are adsorbed to the surface can be tracked across the whole spectrum, which is an additional advantage of plasmonic interferometry compared to more conventional SPR that typically operates at single wavelength. After RCA1 cleaning of the sensor gold surface, the measured peak wavelength of the reference peak is around 588.1 nm (Figure 3i). After each functionalization step, the refractive index near the surface is modified by another layer of biomolecules, resulting in a spectral peak shift towards longer wavelengths. For the APTES and sulfo-NHS-biotin treatment steps (Figure 3ii,iii, respectively) the relative spectral peak shifts compared to their previous steps, are both ∼1.3 nm, while the relative spectral peak shift caused by the addition of streptavidin is ∼2.1 nm (Figure 3iv); finally, surface capture of the biotinylated TREM2 antibody produces an additional shift of ∼1.3 nm. A total spectral peak shift of ∼6 nm was observed after the entire functionalization procedure. The data shown in Figure 3 suggest that plasmonic interferometers can be effectively used to monitor the evolution of complex functionalization steps that involve adsorption of monolayer- and submonolayer-thick molecules to the sensor surface.

## 3. TREM2 Biosensing Experiment: Results and Analysis

### 3.1. Uniformity Study of Surface Functionalization Steps with Plasmonic Interferometry

**Figure 4:**
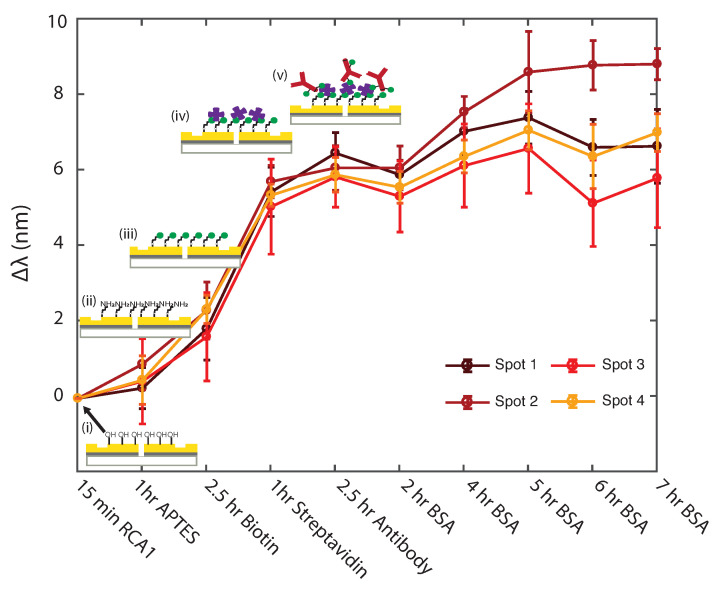
**Uniformity study of surface functionalization steps across four sensing spots.** Circles represent the mean value of wavelength shift (Δλ) measured from 7 GSG plasmonic interferometers after each functionalization step, labelled in the horizontal axis. Error bars represent the standard deviation. Lines and symbols with different colors indicate data measured from different sensing spots.

The sensing chip was functionalized and stored in a refrigerator at 4 °C prior to testing. Immediately before TREM2 detection, the functionalized chip was blocked by 0.5∼1% BSA PBS in order to saturate nonspecific binding sites and prevent false positive results [50]. Figure 4 shows the results of experiments carried out on each of the four sensing spots of the chip, tracking the spectral shift due to each functionalization step and after every hour of BSA blocking. Due to saturation of the blocking agent on the surface over time, the spectral shift due to BSA stabilized, enabling optimization of the BSA blocking time. We estimate that BSA almost saturated the surface of the chip after 4 h of blocking; therefore, the chip was blocked for 7 h at room temperature to ensure complete saturation. After the given blocking time, the chip was rinsed three times with a washing buffer (PBS with 0.05% Tween 20) followed by PBS and DI water, then dried under purified nitrogen gas flow.

### 3.2. Sensing TREM2 Antigen-Antibody Binding Interaction with Plasmonic Interferometry

For sensing experiments, various concentrations of TREM2 in buffer solution were obtained by diluting a stock solution of TREM2 (0.27 mg/mL recombinant Human-TREM2 Fc Chimera PBS (Cat. #BAF1828, R&D)) in 0.5% BSA PBS (0.01 M, pH = 7.4). BSA serves as a protein stabilizer to maintain the integrity of TREM2 at low concentration.

TREM2 binding experiments were performed by directly dispensing 40 μL TREM2 BSA PBS on the chip and then drying it to verify that surface capture and adsorption had effectively taken place. More specifically, after a given binding time interval, the chip was rinsed three times with a washing buffer solution (PBS with 0.05% Tween 20) followed by PBS and then DI water, dried under nitrogen gas flow. Then, transmitted spectra through the slit of each GSG plasmonic interferometer were measured to assess the presence of TREM2 at the sensor surface. This process was repeated to obtain TREM2 kinetic binding curves as a function of time and for various initial TREM2 concentrations in buffer solution.

**Figure 5:**
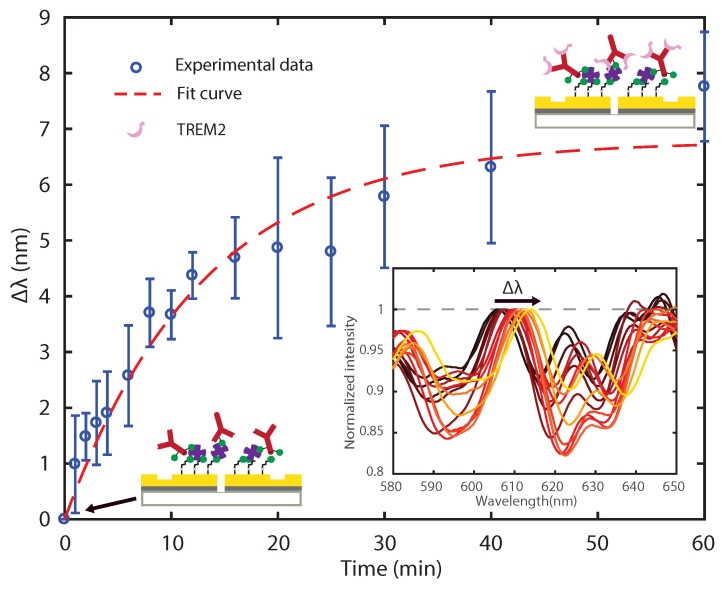
**Sensing temporal evolution of TREM2 surface binding kinetics with plasmonic interferometry.** Blue circles represent the mean peak shift (Δλ) averaged over seven nominally identical GSG plasmonic interferometers as the result of temporal evolution of antigen-antibody binding reaction for a 2.7 ng/ml TREM2 0.5% BSA PBS. Error bars represent the standard deviation. Bottom right inset illustrates the normalized transmitted spectra (averaged over seven identical GSG interferometers) measured at each time step. Color changing from dark red to yellow represents increasing reaction time from 0 to 60 min.

Figure 5 shows the observed peak shift Δλ as a function of time in a binding experiment performed with a 2.7 ng/ml TREM2 0.5% BSA PBS. Blue circles and error bars represent the mean value and standard deviation, respectively, of Δλ measured from seven GSG plasmonic interferometers. The inset of Figure 5 displays the average of normalized intensity spectra across all seven GSG plasmonic interferometers at different time steps, showing a spectral shift towards longer wavelengths as the reaction progresses. The time-domain sensing curve (or “sensorgram”) reported in Figure 5 shows that by tracking the wavelength shift as a function of time we can indeed monitor the capture of TREM2 antigens from antibody binding sites and subsequent formation of a sub-monolayer of TREM2 on the sensor surface.

To understand the kinetic interaction of the binding between TREM2 and its antibody, we first consider an equilibrium model for the chemical reaction [39,51,52,53,54,55,56]. Ideally, the antibody-antigen interaction is a reversible reaction:

(3) antibody+antigen⇌complex.

The time-dependent rate equation that governs this reaction can be expressed by:

(4) $$\frac{d[complex]}{dt}=k_{f}\left[Ab\right]\left[Ag\right]-k_{b}\left[complex\right]$$

where [*complex*] is the molar concentration of antibody-antigen complex, [*Ab*] is the molar concentration of unoccupied antibodies, [*Ag*] is the molar concentration of antigen in the solution, $k_{f}$ is the forward reaction constant and $k_{b}$ is the backward reaction constant. When the reaction reaches equilibrium, we have:

(5) $$k_{f}{\left[Ab\right]}_{eq}{\left[Ag\right]}_{eq}=k_{b}{\left[complex\right]}_{eq}$$

(6) $$K_{d}\equiv \frac{k_{b}}{k_{f}}=\frac{{\left[Ab\right]}_{eq}{\left[Ag\right]}_{eq}}{{\left[complex\right]}_{eq}}$$

where $K_{d}$ is the dissociation constant.

In addition, if we assume that the total number of antibodies immobilized at the sensor surface is fixed and the concentration of antigens is constant due to the large volume (or continuous dispensing) of the sample solution onto the surface, the following expression holds true at all times:

(7) $${\left[Ab\right]}_{0}=\left[Ab\right]+\left[complex\right]$$

where ${\left[Ab\right]}_{0}$ is the initial concentration of the antibodies immobilized on the sensor surface. Applying Equations (6) and (7) to Equation (4) and taking a time integral, we can obtain:

(8) $$\left[complex\right]=\frac{\left[Ag\right]{\left[Ab\right]}_{0}}{\left[Ag\right]+K_{d}}\left[1-e^{-(k_{f}\left[Ag\right]+k_{b})t}\right].$$

Equation (8) implies that: (a) the concentration of the antibody-antigen complex at the surface increases exponentially as a function of time; (b) the concentration of the antibody-antigen complex at equilibrium is always lower than the initial concentration of antibodies immobilized at the sensor surface; and (c) by increasing the antigen concentration in solution, the binding kinetics should occur with a faster rate and the equilibrium complex concentration should also increase. Figure 5 reports an exponential fit of the experimental data, where we assumed that the relative peak shift Δλ was directly proportional to the number of TREM2 (the antigen) proteins captured by the immobilized antibodies and binding at the sensor surface over time. The data were fit by $\Delta \lambda =a(1-e^{-t/\tau })$, where a=7.12 nm and τ=13.44 min. Several studies have shown that protein adsorption is a very sophisticated process, strongly influenced by experimental parameters, such as surface wettability, pH, protein structure, and other factors [57,58]. Moreover, proteins could be adsorbed to the surface in a multilayer fashion, especially on a hydrophobic surface, which would produce a higher (and therefore more readily detectable) Δλ than that caused by the formation of a single monolayer.

**Figure 6:**
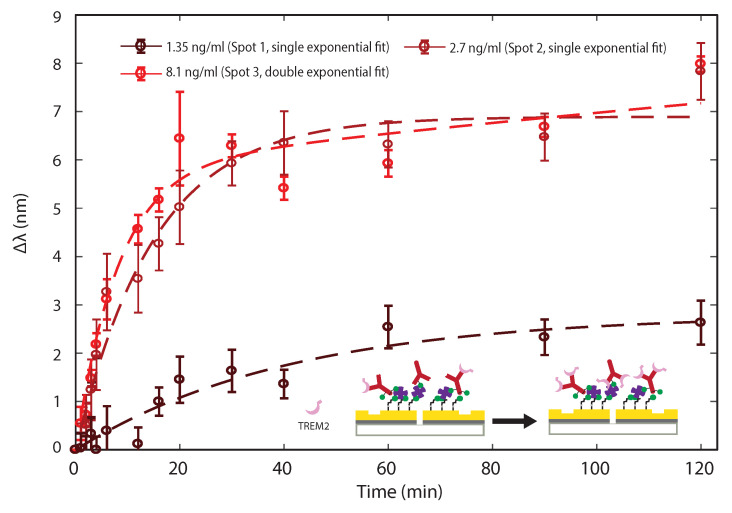
**Binding times for different TREM2 concentrations.** Temporal evolution of peak wavelength shifts measured from normalized transmission spectra for different TREM2 concentrations. Error bars represent standard deviation from 7 GSG interferometers. Dashed lines represent exponential fit using the kinetic model provided in the text.

To further validate the model, we performed sensing experiments to detect TREM2 in solution with three different physiological concentrations (1.35 ng/ml, 2.7 ng/ml, and 8.1 ng/ml) by using three of the four sensing spots of the chip, separately, and investigated the concentration dependence of the binding kinetics. Note that the chip was first regenerated to bare gold by 15 min of RCA1 cleaning, then functionalized and blocked again with BSA PBS before TREM2 detection. After regeneration, we confirmed that the normalized transmission spectra went back to the initial spectra, well within the confidence intervals reported in Figure 3. Figure 6 shows the sensing results averaged over the three functionalized sensing spots after interaction with 0.5% BSA PBS spiked with different TREM2 concentrations. The Δλ associated with the binding reaction corresponding to 1.35 ng/ml and 2.7 ng/ml TREM2 concentration showed a single-exponential time dependence $\Delta \lambda =a(1-e^{-t/\tau })$, where a=2.8 nm, τ=41.08 min for 1.35 ng/ml and a=6.89 nm, τ=15.04 min for 2.7 ng/ml, which is consistent with the parameters identified for the same concentration in Figure 5. When the concentration of TREM2 increased from 1.35 ng/ml to 2.7 ng/ml, the binding time constant τ decreased, which confirms that the reaction occurred faster in the presence of a higher concentration of antigens, as predicted by the model. Additionally, the parameter *a*, which represents the total wavelength shift at equilibrium, increased with the concentration of antigens. These experimental findings are consistent with the simple model described by Equation (8).

Interestingly, the binding reaction data corresponding to the higher concentration (8.1 ng/ml TREM2) displayed a kinetic curve that has a second slow rising stage, which could be fit with a double-exponential function: $\Delta \lambda =a_{1}\left(1-e^{-t/\tau_{1}}\right)+a_{2}\left(1-e^{-t/\tau_{2}}\right)$, where $a_{1}=5.83$ nm, $\tau_{1}=8.1$ min, $a_{2}=6.08$ nm, and $\tau_{2}=476.19$ min. This could indicate the presence of multiple adsorption mechanisms with distinct dissociation constants $K_{d}$ that were active at higher analyte concentrations. These different values of $K_{d}$ were manifested in the double-exponential curve by unique binding time constants $\tau_{1}$ and $\tau_{2}$ and associated wavelength shifts $a_{1}$ and $a_{2}$. Previous studies reported similar two-stage adsorption kinetics, which may be due to a number of possible mechanisms. For instance, the initial stage may be caused by formation of a single monolayer at the surface, while the second slow rising stage could correspond to multilayer condensation of proteins on the surface [57,59]. The longer time constant may also arise from more subtle adsorption mechanisms the various proteins may be subjected to: for example, for BSA, conformational changes may be the primary reason for multilayer conformation, while for the TREM2 antibody (immunoglobulin G), multilayer conformation behavior could be significantly influenced by long-range electrostatic interaction [60]. In addition, higher-order interaction effects during the adsorption process could cause the combined kinetics to differ significantly from a summation of two single component adsorption kinetics [61,62], especially at higher analyte concentrations.

Although a more complex model may be needed to fully describe all of the possible microscopic phenomena underlying the actual reaction kinetics, to first approximation the data presented in Figure 6 can be fit using the simple exponential model which allows us to generate reliable calibration curves that accurately describe the proposed sensing mechanism. For instance, by choosing a given incubation time of t=10 min, the wavelength shifts observed at three different concentrations are statistically different, enabling us to infer the concentration based on the observed Δλ. However, we believe that a fit to the full time-resolved data set is an overall more statistically significant analysis of the acquired data and can lead to a better discrimination between different analyte concentrations, as shown in Figure 6. The full set of data presented so far validates the possibility of using plasmonic interferometry coupled with surface functionalization as a viable sensing scheme for TREM2 detection in solution.

## 4. Conclusions

We demonstrate a biophotonic sensing platform to monitor the chemical reactions associated with surface functionalization of a metal in time series through wavelength shifts in the transmission spectra of plasmonic interferometers. This work helps us devise and assess a functionalization protocol for detecting TREM2, a biomarker associated with the development of AD and other neurodegenerative diseases, in solution. We devise a chemical equilibrium model which we fit with the reported data. The fitting parameters from the data reinforce the theoretical model and are consistent between experiments, suggesting that the experimental process is repeatable. The chemical equilibrium model allows us to generate calibration curves for the reported data which are able to differentiate between different concentrations of TREM2. The results reported here open up the possibility to employ the proposed sensing platform based on plasmonic interferometry for the detection of physiological concentrations of TREM2 in CSF or other bodily fluids. In the future, plasmonic interferometry may be a promising method to test functionalization protocols for deployment on other scalable platforms, such as colloidal nanoparticles, which are widely used in biological testing, to help validate the importance of TREM2 to the development pathway of AD. Additionally, plasmonic interferometry may be adapted to other surface functionalization protocols for different antibody-antigen pairs to enable development of a wide array of testing schemes for a variety of clinically relevant biomarkers.

## Data Availability

The data presented in this study are available on request from the corresponding author.

## Abbreviations

The following abbreviations are used in this manuscript:

AD	Alzheimer’s disease
APTES	(3-Aminopropyl)triethoxysilane
BSA	Bovine serum albumin
CCD	Charged-coupled device
CSF	Cerebrospinal fluid
DI	Deionized
ELISA	Enzyme-linked immunosorbent assay
FIB	Focused ion beam
GSG	Groove-Slit-Groove
NA	Numerical aperture
NHS	N-Hydroxysulfosuccinimide
PBS	Phosphate buffer solution
RCA1	Radio Corporation of America Si wafer cleaning procedure, standard clean-1
SEM	Scanning Electron Micrograph/Microscopy
SPP	Surface Plasmon Polariton
SPR	Surface Plasmon Resonance
TREM2	Triggering Receptor Expressed on Myeloid Cells 2
